# The impact of glycolysis on ischemic stroke: from molecular mechanisms to clinical applications

**DOI:** 10.3389/fneur.2025.1514394

**Published:** 2025-01-24

**Authors:** Yingquan Liu, Peijia Hu, Hongliang Cheng, Fangyuan Xu, Yu Ye

**Affiliations:** ^1^The First Clinical College of Anhui University of Chinese Medicine, Hefei, China; ^2^The Second Affiliated Hospital of Anhui University of Chinese Medicine, Hefei, Anhui, China; ^3^Graduate School, Anhui University of Chinese Medicine, Hefei, Anhui, China

**Keywords:** ischemic stroke, glycolysis, glucose metabolism, pathomechanism, diagnosis, treatment

## Abstract

Ischemic stroke (IS), a leading cause of disability and mortality worldwide, remains a significant challenge due to its complex pathogenesis. Glycolysis, a central metabolic pathway, plays a critical role in bridging the gap between metabolic dysfunction and neurological impairment. During ischemic conditions, glycolysis replaces oxidative phosphorylation as the primary energy source for brain tissue. However, in the ischemia–reperfusion state, neuronal cells show a particular reliance on aerobic glycolysis. Immune cells, such as monocytes, also contribute to atheromatous plaque formation and thrombi through increased aerobic glycolysis. Given glycolysis’s involvement in various pathological stages of IS, it offers the potential for improved diagnosis, treatment, and prevention. This review comprehensively explores the role of glycolysis in different phases of IS, addresses existing controversies, and discusses its diagnostic and therapeutic applications. By elucidating the intricate relationship between glycolysis and IS, this review aims to provide novel insights for future research and clinical advancements.

## 1 Introduction

Stroke, a prevalent cerebrovascular disease characterized by sudden onset neurological deficits, is a significant global health concern. Primarily caused by damage to cerebral blood vessels, strokes result in focal or diffuse brain tissue injury. With over 10 million new cases and 6.55 million deaths annually, stroke ranks as the second leading cause of death worldwide, surpassed only by ischemic heart disease (1–3). The enduring impact of stroke extends beyond mortality, as up to 50% of survivors experience long-term disability, making it the third leading cause of disability globally (4, 5). Strokes are categorized into ischemic strokes (IS), resulting from disrupted blood supply to the brain, and hemorrhagic strokes, caused by bleeding from a ruptured blood vessel. Ischemic strokes account for approximately 85% of all stroke cases (6).

The underlying pathological mechanism of IS is the formation of thrombus in blood vessels. This leads to disruption of the blood supply to an area of the brain, leading to localized necrosis of brain tissue and neuronal damage. Three main factors are known to cause IS: (1) Cerebrovascular atherosclerotic plaques and their rupture (in nearly half of the cases); (2) Cardiogenic cerebral infarction (in about 20% of the cases); (3) Cavernous cerebral infarction caused by small-vessel lesions (in about 20% of the cases) (7). In addition, other factors, such as vasculitis, extracranial arterial entrapment, and pathogenic infections, can lead to IS (8, 9). Current management of acute ischemic stroke (AIS) primarily involves recombinant tissue plasminogen activator (rt-PA) intravenous thrombolysis and/or mechanical thrombectomy (10). While these approaches can be practical, their success is limited to a small proportion of patients due to narrow time windows and other factors (11). The urgent need for innovative therapeutic strategies to address ischemic stroke is underscored by these limitations.

The primary metabolic pathways for glucose sugars include glycolysis, the pentose phosphate pathway (PPP), and oxidative phosphorylation (OXPHOS). Under normal conditions, the brain primarily relies on OXPHOS for energy, accounting for approximately 95% of the ATP required to maintain its function (12). However, in AIS, OXPHOS is inhibited, leading to disrupted ATP production and glucose metabolism. During this period, neurons predominantly depend on anaerobic metabolic processes like glycolysis for energy (13, 14). Glycolysis degrades glucose and glycogen into pyruvate, generating ATP. In the presence of adequate oxygenation, pyruvate can produce significant amounts of ATP through the tricarboxylic acid (TCA) cycle. Nevertheless, during hypoxia or periods of high energy expenditure, pyruvate is reduced to lactate (15). While excessive lactic acid accumulation in ischemic brain tissue can contribute to increased neurological damage (15, 16). Recent studies have demonstrated that lactate can have a protective effect on mouse neurons during ischemia and hypoxia in AIS (17).

Glycolysis is a metabolic pathway primarily occurring under anaerobic or hypoxic conditions (18). Notably, during ischemia–reperfusion (IR), when blood oxygenation to ischemic tissue is restored, neurons exhibit a preference for glycolysis as their primary energy source, even in the presence of adequate oxygen supply (19, 20). This metabolic shift in glucose utilization was initially observed in cancer cells and is referred to as the Warburg effect (also known as aerobic glycolysis, which involves the conversion of glucose to lactate in the presence of oxygen) (21, 22). While previous studies suggested that aerobic glycolysis following IR might have detrimental effects on neurons, including exacerbated neuroinflammation and enhanced nerve damage (23, 24). Recent research has revealed that enhanced aerobic glycolysis can be protective for nerve cells and promote nerve damage repair in a mouse model of focal ischemic stroke (25).

The role of glycolysis in the pathophysiology of IS remains a subject of ongoing debate. This review aims to comprehensively examine recent research on glycolysis and IS, exploring its involvement in the progression of ischemic stroke, its diagnostic value, and its potential therapeutic applications. We will also address current controversies and offer our perspective. By synthesizing this information, we hope to provide valuable insights for future scientific research and clinical approaches to the diagnosis and treatment of IS.

## 2 The pathogenesis of ischemic stroke

The pathogenesis of IS remains incompletely understood. However, current research suggests it primarily involves inflammatory responses, oxidative stress, excitotoxicity, apoptosis, and other related factors (26). Following IS, focal ischemia triggers a robust inflammatory response, primarily mediated by the activation of microglia and astrocytes (27). These cells subsequently release pro-inflammatory mediators such as tumor necrosis factor-*α* (TNF-α), interleukin-1β (IL-1β), interleukin-6 (IL-6), and matrix metalloproteinases (MMPs) from injured tissues, which in turn promote the overexpression of genes involved in inflammation, neurotoxicity, and glycolysis (28, 29). Notably, this is different from the inflammatory response caused by IS due to pathogen infection. Bacterial virulence factors and pathogens directly breach the blood–brain barrier, subsequently triggering leukocytes to respond to infection (9). Cytokines released in this way cause a series of immune injuries called pathogen-associated molecular patterns (PAMPs). Metabolism-related sterile inflammation induced by damaged tissue-derived danger signals after stroke onset is termed damage-associated molecular patterns (DAMPs) (30). The high-mobility box group 1 (HMGB1) is recognized as the representative DAMPs (31). It is released upon cell and tissue necrosis and is actively produced by immune cells (32). In the core region of cerebral infarction, severe ischemia and hypoxia-induced metabolic disturbances lead to an increased release and impaired reuptake of glutamate, the primary excitatory neurotransmitter of the nervous system. This excessive glutamate accumulation in ischemic brain regions subsequently activates the glutamate-gated ion channel N-methyl-D-aspartate receptor (NMDAR), resulting in the influx of calcium and the activation of downstream cell death signaling pathways. This, in turn, leads to the aberrant activation of numerous calcium-dependent enzymes in the brain, initiating neuronal necrosis, apoptosis, and autophagy processes (33). During cerebral IR, neuronal mitochondrial dysfunction and excessive reactive oxygen species (ROS) accumulation generate oxidative stress and release large amounts of free radicals (e.g., nitric oxide (NO), peroxynitrite), further exacerbating excitotoxicity and leading to neuronal apoptosis and death (26).

## 3 Glial cells in ischemic stroke

**Figure 1:**
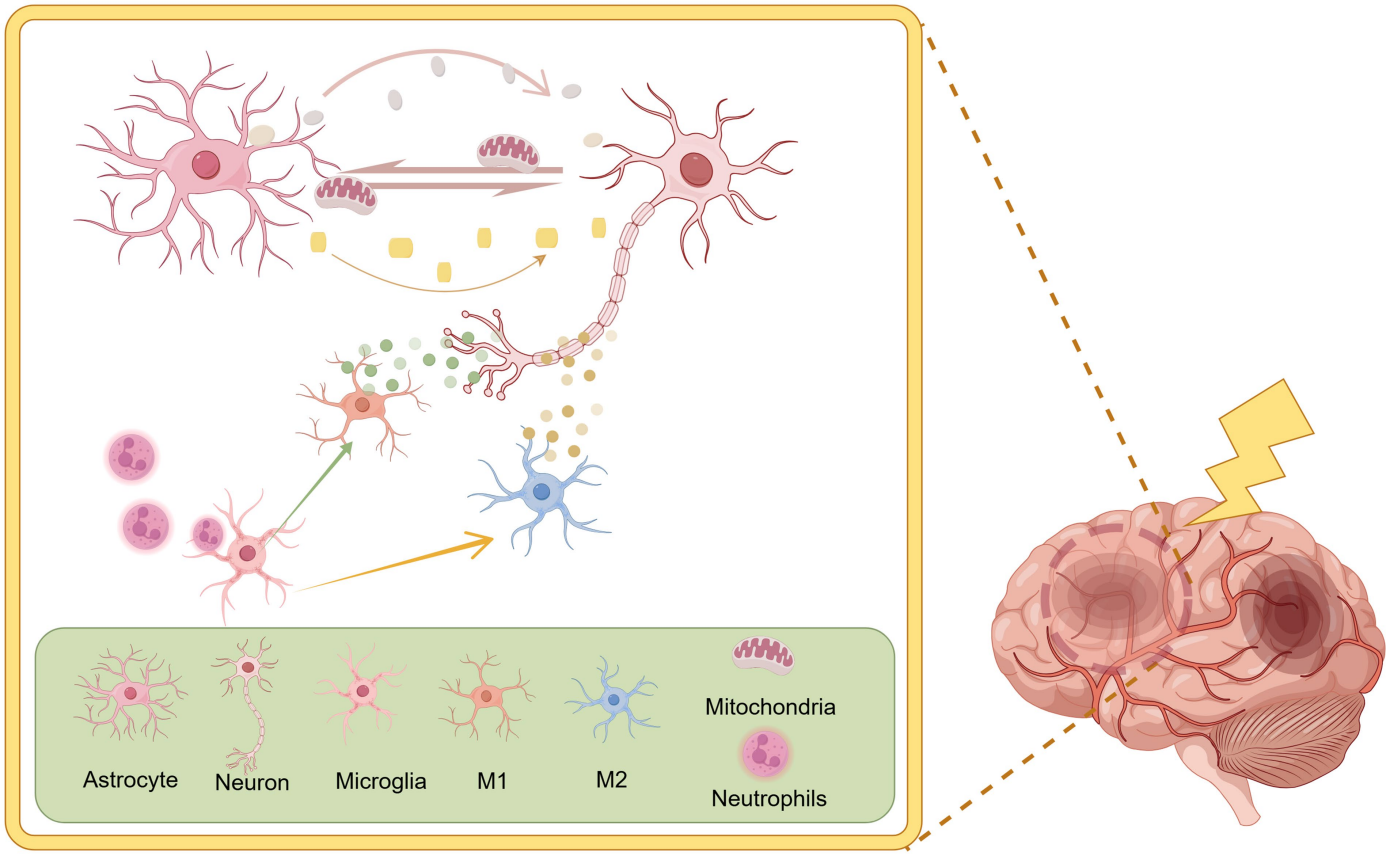
There is a complex regulatory relationship between neurons and glial cells. Activated microglia can differentiate into pro-inflammatory phenotypes (M1) and anti-inflammatory phenotypes (M2). Microglia also possess immunophagocytic and surveillance functions, resulting in different regulatory effects on neurons. Astrocytes have been shown to facilitate the transfer of energy and antioxidants to neurons, thereby providing crucial support for their function. By Figdraw.

In the brain, nerve cells are divided into two main categories: neurons and glial cells. Glial cells are further divided into astrocytes, microglia, and oligodendrocytes (34). They all play a crucial role in the course of IS. However, glial cells may have either protective or damaging effects on neurons in IS (given the relative paucity of studies of oligodendrocytes in IS and glycolysis, they will not be addressed in this review). Figure 1 summarises the main relationships between neurons and glial cells.

Microglia are the main nerve cells in the brain responsible for immunity and are important for normal brain functioning (35). During the initiation and progression of acute IS, the neuroinflammatory response induced by microglial activation is of primary concern (36). Activated microglia exhibit two distinct phenotypes. One is the pro-inflammatory type (M1), which triggers neuroinflammatory responses and tissue damage by secreting pro-inflammatory factors. The other is the anti-inflammatory type (M2), which releases anti-inflammatory factors to provide protection to nerves and promote the repair of damaged tissues (37). Microglia in IS usually present an M1 phenotype, a state in which they have a destructive effect on neurons and are detrimental to the recovery of brain tissue functions (38). Besides, microglia have immunosurveillance and phagocytosis functions and are responsible for removing pro-inflammatory cells that are over-activated in IS (39).

Astrocytes are also the major glycogen storage units in the brain. Physiologically they consume large amounts of glucose and produce lactate. Strong aerobic glycolysis is present regardless of oxygen availability (29, 40). Thus, compared to neurons, astrocytes exhibit greater tolerance to nutrient depletion and oxidative stress (29). Reactive astrocytes (activated astrocytes) after IS onset rely on glycolysis to survive in large numbers and to play a regulatory role (41). The survival of neuronal cells after IS onset is closely related to the regulatory role of astrocytes. This regulatory role is mainly reflected in the following aspects: (1) Glycolytic processes are increased in morpho glial cells during hypoxia, producing small concentrations of lactate that are delivered to neurons for their utilization (42). This process is called the astrocyte-neuron lactate shuttle (ANLS) (43). (2) Neurons can release damaged mitochondria and transfer them to astrocytes for processing, followed by the release of healthy mitochondria used to maintain the health of the holding neuron (44). However, when ischemic damage is severe, astrocytes also release damaged mitochondria to neurons, which in turn affects neuronal health (45). (3) Glutathione (GSH) concentration in astrocytes is twice that of neurons and maintains neuronal resistance to oxidative stress during ischemia and hypoxia (29). (4) Reactive astrocytes release neuroprotective agents during the onset of cerebral ischemia. Whereas overproliferating astrocytes form a scar in the later stages of cerebral ischemia and physically impede recovery from neurological injury (46).

How to regulate the phenotypic transition of microglia and the functional role of astrocytes on neurons. Thus, utilization of these cells with dual roles has become the focus of current research.

## 4 Glycolysis is involved in the development of ischemic stroke

It is well-established that impaired glucose and oxygen delivery during cerebral ischemia disrupts oxidative phosphorylation. Consequently, the brain rapidly transitions its primary metabolic strategy from oxidative phosphorylation to glycolysis to fulfill the energy demands of the ischemic region. However, the increased glycolysis during cerebral ischemia has been associated with detrimental effects on salvageable tissue survival, as it can lead to lactate accumulation and ROS production (47). This phenomenon is prevalent in both clinical and experimental settings, particularly in stroke patients with hyperglycemia (16).

There is an inter-regulatory relationship between glucose metabolism and the immune system (48, 49). Aerobic glycolysis was found to be an important factor in activating microglia and shifting them to the M1-polarized state, promoting their pro-inflammatory effects (24, 50). In addition, there is evidence that microglia phagocytosis is critical for counteracting neutrophil tissue damage during the reperfusion period of IS. Microglia will preferentially utilize aerobic glycolysis for energy supply over OXPHOS after reperfusion. Reduced microglial phagocytosis due to a relative lack of ATP supply (51). Overproliferation of reactive astrocytes after ischemia leads to the production of glial scar. Glycolysis may accelerate this process (46, 52).

It is now understood that glutamate- or NMDA-mediated excitotoxicity triggers neuronal death by stimulating the expression of relevant apoptotic proteins (53). This process is accompanied by the activation of glycolysis (54). In ischemic tissues, excessive accumulation of calcium ions in mitochondria leads to depolarization and dysfunction of their membranes (53). Mitochondrial autophagy is crucial for maintaining neuronal bioenergetic homeostasis by effectively removing damaged mitochondria. However, highly active glycolysis following ischemia can disrupt this repair mechanism (55).

The aerobic glycolysis pathway is no longer exclusively associated with cancer cells. Emerging evidence suggests that stem cells, immune cells, and other cell types also utilize aerobic glycolysis for their physiological or pathological functions (56). Neural progenitor cells (NPCs) have been shown to have limited capacity for aerobic glycolysis in hypoxic environments, leading to their preferential differentiation into glial cells (57). However, by inhibiting aerobic glycolysis, NPCs can be effectively promoted to utilize pyruvate for energy production and drive the oxidative phosphorylation process, thereby favoring their differentiation toward neurons (58). This suggests a potential role for aerobic glycolysis in nerve damage repair after stroke. Additionally, aerobic glycolysis is significantly involved in the formation of atherosclerotic plaques and arterial and venous thrombi through its regulation of immune cell proliferation and activation, primarily involving monocytes/macrophages, neutrophils, and platelets (39, 59–62). Consequently, aerobic glycolysis profoundly influences the probability of ischemic stroke occurrence and recurrence.

## 5 Enzymes and metabolites related to glycolysis in ischemic stroke

**Figure 2:**
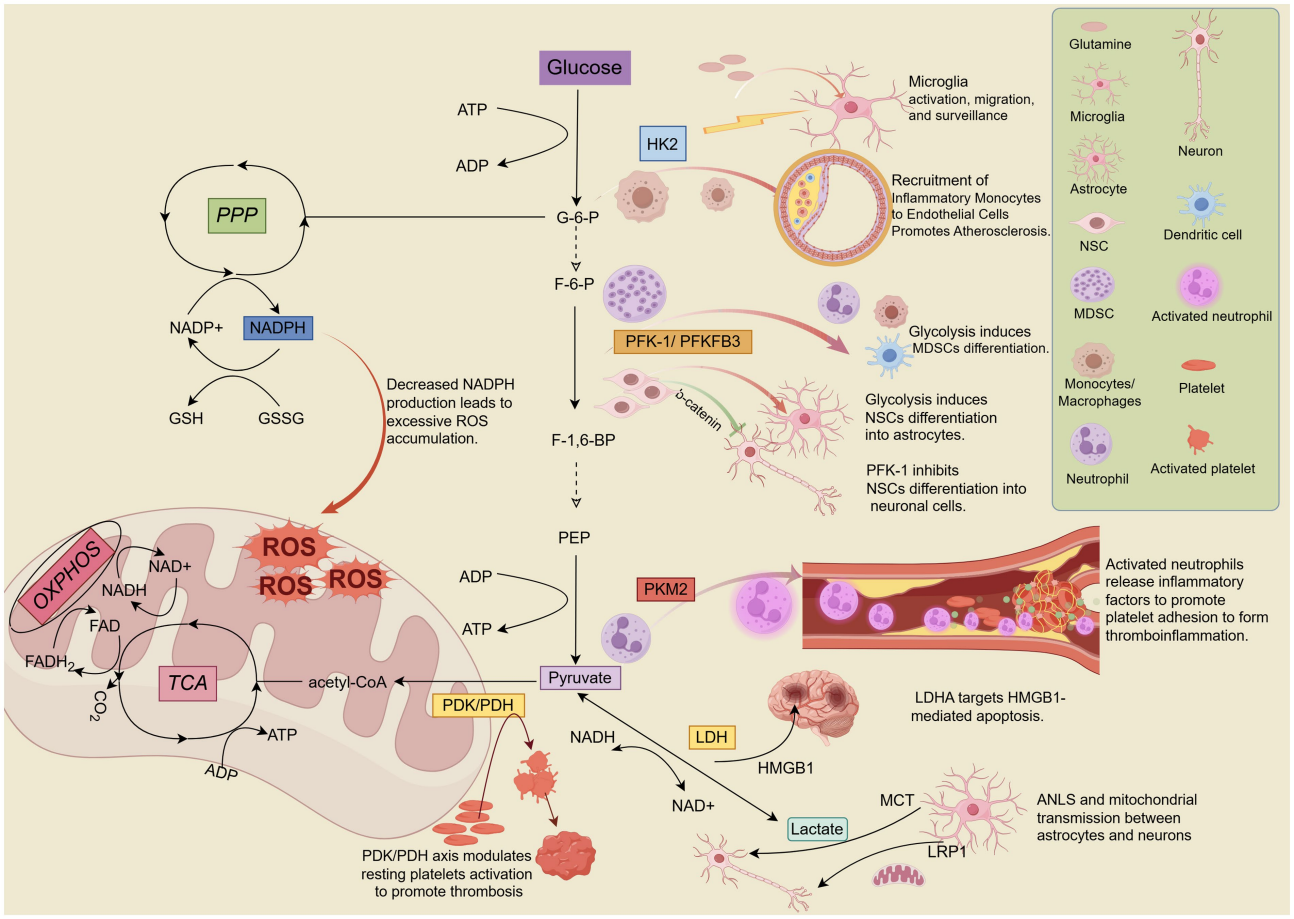
Hexokinase 2 (HK2), phosphofructokinase-1/6 (PFK-1/PFKFB3), pyruvate kinase M2 (PKM2), pyruvate dehydrogenase kinase/pyruvate dehydrogenase (PDK/PDH), and lactate dehydrogenase A (LDHA) are key enzymes and kinases involved in the regulation of glycolysis. These enzymes play crucial roles in the pathophysiological processes of IS, including neuroinflammation, mitochondrial oxidative stress, neuronal differentiation, apoptosis, and others. Lactate, the end product of glycolysis, directly or indirectly influences lactate shuttling and mitochondrial transport between neurons and astrocytes, contributing significantly to the progression of pathological mechanisms in IS. By Figdraw.

The glycolytic pathway is regulated by three key rate-limiting enzymes: Hexokinase (HK), Phosphofructokinase (PFK), and Pyruvate kinase (PK), which catalyze irreversible reactions (18). While pyruvate production marks the end of the glycolytic pathway, the process does not necessarily cease. Lactate dehydrogenase (LDH) subsequently regulates the reversible conversion of pyruvate to lactate (63). Although pyruvate dehydrogenase (PDH) and pyruvate dehydrogenase kinase (PDK) are located in the mitochondria, they influence glycolysis by regulating the flux of pyruvate into the TCA cycle, which in turn affects glycolytic activity (64). Growing evidence suggests that these enzymes and their products play significant roles in the pathophysiological processes of IS through complex regulatory mechanisms. Figure 2 summarises the contributions of glycolysis-related enzymes and products to IS.

### 5.1 HK2

Hexokinase is the initial enzyme in the glucose metabolic pathway and the first key rate-limiting enzyme in glycolysis. It catalyzes the phosphorylation of glucose, consuming ATP to produce glucose-6-phosphate (G-6-P) (65). The formation of G-6-P initiates the major pathways of glucose utilization: glycolysis, PPP, and OXPHOS. Therefore, HK is considered a key regulator of glucose metabolism (66). Mammalian tissues express five hexokinase isozymes: HK1, HK2, HK3, HK4, and HK structural domain component 1 (HKDC1), each with distinct tissue distribution, biochemical profiles, and catalytic activities (67, 68). HK1 is widely expressed in mammalian tissues, particularly the brain, and is closely linked to the activation of inflammatory pathways in neurological diseases. It is also known as “brain hexokinase” but is not significantly regulated by most physiological, hormonal, and metabolic factors (68–70). While HK2 has limited expression in most normal tissues, it is frequently overexpressed in various cancer tissues and has recently been found to be overexpressed in ischemic stroke brain tissues (68, 71, 72). HK3 is highly expressed in bone marrow, lung, liver, and kidney (73). HK4 is predominantly expressed in hepatic and pancreatic *β*-cells, regulating insulin secretion and hepatic glycogen synthesis and catabolism (74, 75). HKDC1, primarily expressed in the kidney and intestine, is a novel HK isoform most similar to HK1 (76). Notably, HK4 has only one kinase structure active site domain with enzymatic activity, whereas HK1, HK2, and HK3 possess two kinase structurally active site domains (n- and c-domains). While both domains of HK1 and HK2 are enzymatically active only at the c-terminus, HK2’s n- and c-terminal domains are both enzymatically active (74). This confers a higher glucose affinity and catalytic efficiency to HK2 than other HK isoforms. Interestingly, HK2’s unique n-terminal catalytic structural domain, which binds to the voltage-dependent anion channel (VDAC) of the mitochondrial outer membrane, enables it to further regulate cell activity and apoptosis (77). Additionally, HK2 is the most potent isoform in promoting aerobic glycolysis among all HK isoforms (78). These properties suggest that HK2 plays a unique role in glucose metabolism under various physiological and pathological conditions and may be a significant regulated isoform in cells of a wide range of tissues.

Neuroinflammatory responses are a prominent pathological feature of ischemic stroke during acute ischemia and/or reperfusion (79). Cell culture studies have demonstrated that microglia-mediated neuroinflammation under hypoxic conditions requires elevated glycolysis and HK2 induction. Upregulation of HK2 leads to the accumulation of acetyl-coenzyme A, contributing to histone acetylation and IL-1β transcriptional activation. However, inhibiting HK2 *in vivo* has been shown to prevent microglia activation, IL-1β production, and ischemic brain injury (80). It is generally believed that inhibiting HK2 can reduce glycolytic flux, potentially alleviating immune cell activation and pathological processes in inflammatory diseases (49). Interestingly, a recent study found that HK2 knockdown exacerbated neuroinflammation and worsened brain injury in a photothrombotic stroke model. This was attributed to HK2 knockout inhibiting microglia’s glycolytic flux and energy production, limiting their proliferation, and reducing surveillance and injury-triggered migration (72). However, other studies have shown that microglia are metabolically flexible and can rapidly adapt to utilize glutamine as an alternative energy source to support their immunosurveillance functions in the brain parenchyma, even when glucose is limited (81). Besides, inhibition of aerobic glycolysis in microglia in a mouse model of middle cerebral artery occlusion promoted glutamine solubilization in microglia, thereby providing fuel for OXPHOS (51). It has also been reported that glutamine utilization also promotes macrophage phagocytosis and M2-type activation, exerting anti-inflammatory effects (82, 83). The discrepancies in these findings may be related to the different models used and the adoption of various interventions, warranting further investigation. Atherosclerosis is a chronic inflammatory disease of the vascular wall (84). It is not only a common cause of stroke but also a significant risk factor for recurrent stroke (85). Initially, monocytes invade and accumulate in the dilated arterial wall, differentiating into macrophages. The pro-inflammatory processes mediated by these macrophages are the primary drivers of plaque formation. Macrophage proliferation is another critical factor in further plaque growth (84, 86). The study reveals that levels of HK2 are significantly increased in monocytes from patients with IS as well as IS model mice. HK2 exacerbated vascular inflammation and accelerated the process of atherosclerosis by promoting the recruitment of inflammatory monocytes to endothelial cells and upregulating Il-1β expression (59).

### 5.2 PFK-1/PFKFB3

Phosphofructokinase is the second key rate-limiting enzyme in glycolysis, catalyzing the irreversible conversion of fructose-6-phosphate (F-6-P) to fructose-1,6-bisphosphate (F-1,6-BP), a crucial control point in regulating glycolytic flux (87). PFK-1 is a tetrameric protein encoded by three genes: PFK-M (muscle), PFK-L (liver), and PFK-P (platelet or brain) (88, 89). The activity of PFK-1 is modulated by various cytoplasmic substances, including adenosine triphosphate (ATP), adenosine diphosphate (ADP), F-6-P, and fructose-2,6-bisphosphate (F-2,6-BP). Among these, F-2,6-BP is the most potent allosteric activator of PFK-1, regulating increased glycolytic flux (90). The synthesis and degradation of F-2,6-BP are catalyzed by the bifunctional enzyme 6-phosphofructose kinase/fructose-2,6-bisphosphatase (PFKFB). PFKFB possesses both kinase and phosphatase activities, with the kinase domain located at the N-terminus and the bisphosphatase domain at the C-terminus. Despite an 85% overlap in the core sequences of these domains, they exhibit distinct functional properties due to differences in surrounding residues and the active site (87, 90, 91). The four genes encoding PFKFB isoforms, *PFKFB1*, *PFKFB2*, *PFKFB3*, and *PFKFB4*, exhibit varying kinase/phosphatase activities and tissue expression profiles (87). PFKFB1 is predominantly expressed in liver, muscle, and fetal tissues. PFKFB2 is mainly found in the heart, kidney, and specific cancer cells. PFKFB3 is predominantly expressed in the brain, placenta, and adipose tissue and is also the most abundant isoform in proliferating cells and cancer cells. PFKFB4 is primarily distributed in the testis but also in various tumor cells (87, 92). Notably, among the PFKFB members, PFKFB3 exhibits a significantly higher kinase/phosphatase ratio (730/1), representing more significant enzymatic activity. This increased sensitivity for F-2,6-BP production allows PFKFB3 to tightly control the rate of glycolysis under both normal and pathophysiological conditions (91, 93).

Myeloid-derived suppressor cells (MDSCs) are a heterogeneous population of immature myeloid cells characterized by immunosuppressive properties and elevated glycolytic activity in inflammatory diseases (49). However, MDSCs can differentiate into mature inflammatory cells (neutrophils, macrophages, dendritic cells) with pro-inflammatory effects (94). There is evidence that MDSC significantly accumulated in acute IS patients with ischemic stroke mouse models. Ischemic brain injury can be ameliorated by knockdown or inhibition of PFKFB3 is able to block MDSC differentiation and increase endogenous MDSCs, thereby suppressing the activation state of T effector cell subpopulations (95). Glycolysis is not only closely linked to the maturation and differentiation of immune cells but also plays a crucial role in the differentiation of neural stem cells (NSCs). NSCs are typically found in the dentate gyrus and subventricular zone of the mammalian hippocampus and maintain lifelong neurogenesis by proliferating and producing neurons and glial cells, contributing to the repair of brains damaged by neurodegenerative diseases and/or ischemic injury (96–98). Studies have shown that NSCs differentiation and maturation are marked by a transition from aerobic glycolysis to oxidative phosphorylation (58, 98). The availability of oxygen and the shift in energy metabolism influence cell proliferation and phenotypic differentiation (99). A recent study found that inhibiting PFK-1 expression enhanced neuronal differentiation and dendritic development, improving spatial memory recovery after stroke. Interestingly, these effects of PFK-1 are closely associated with its promotion of *β*-catenin nuclear translocation and activation of downstream signaling, independent of the Wnt signaling pathway (100). Normally, differentiated mature neurons maintain neuronal redox status through the PPP with low PFKFB3 abundance (101, 102). However, during cerebral IR, neuronal redox homeostasis is disrupted due to a shift from the PPP pathway to aerobic glycolysis, accompanied by a significant increase in PFKFB3 expression and a decrease in NADPH production, an essential cofactor for reducing glutathione disulfide (55). This leads to neuronal mitochondrial dysfunction, excessive ROS accumulation, increased oxidative stress, excitotoxicity, and ultimately, neuronal apoptosis and death (20, 102). Inhibiting PFKFB3 activity in an *in vitro* IR model blocks glycolytic activation, forcing neurons to switch to the PPP metabolic pathway, thereby realizing a neuroprotective effect (54).

### 5.3 PKM2

Pyruvate kinase is the key rate-limiting enzyme in the final step of the glycolytic pathway, catalyzing the conversion of phosphoenolpyruvate (PEP) to phosphoenolpyruvate (ADP) to produce pyruvate and ATP (103, 104). In mammals, PK is encoded by two distinct genes, *PKLR* and *PKM*, which can generate two different isoforms. This results in four distinct isoforms: PKR, PKL, PKM1, and PKM2 (105). The expression of these PK isoforms is tissue-specific, influenced by the tissue’s energy requirements and nutrient availability (106, 107). PKM1 is predominantly expressed in adult tissues with high ATP demands, such as the heart, skeletal muscle, and brain (105). PKM2 is expressed in tissues at all stages of embryonic development (108). However, in most cases, PKM2 is gradually replaced by other PK isoforms during tissue growth (109). While PKM2 is primarily expressed in embryonic tissues, it has also been found in other tissues, including malignant tumors, lungs, kidneys, liver (110, 111) and various cell types such as stem cells, neuronal cells, endothelial cells, platelets, neutrophils, macrophages, T cells, etc. (39, 62, 112–116). Notably, PKM1 is the most abundantly expressed isoform in neuronal cells under normal physiological conditions, whereas PKM2 is relatively less expressed. PKM2 is primarily expressed in embryonic cells and NPCs in the hippocampus, cerebellum, and subventricular region (117). Furthermore, PKM2 is overexpressed in various cancers, conferring a growth and survival advantage to cancer cells and promoting tumor cell proliferation and metastasis (118). Beyond its classical functions, emerging evidence suggests that PKM2 plays a significant role in neurological disorders (105).

PKM2 is a complexly regulated protein involved in multiple signaling pathways. In addition to regulating glycolysis and promoting tumor cell proliferation, PKM2 also has biological functions in regulating inflammatory responses, oxidative stress, apoptosis, and mitochondrial dysfunction (105, 119). Leukocytes, represented by neutrophils in ischemic tissues, are recruited by cell adhesion molecules on endothelial cells, leading to tissue inflammatory infiltration damage. Simultaneously, platelets adhere and are activated at the site of ischemic stroke vascular lesions, increasing the risk of secondary thrombotic events. The intricate relationship between thrombosis and inflammatory pathways during stroke pathology has shaped the concept of thromboinflammation (79, 120–122). PKM2 is a modulator of systemic inflammation (123). Nuclear PKM2 is upregulated in peripheral neutrophils after ischemic stroke episodes in humans and mice, resulting in their hyperactivation (39). It was found that the expression of inflammatory factors (TNF-*α*, IL-1β, and IL-6) in neutrophils after cerebral ischemia or reperfusion was significantly reduced in mice with deletion of the PKM2 gene in myeloid cells (39). Additionally, medulla-specific PKM2-deficient mice exhibited improved local cerebral blood flow, reduced infarct size, and enhanced long-term sensorimotor recovery. This is attributed to PKM2’s involvement in post-ischemic cerebral thrombotic inflammation through its regulation of fibrinogen, platelet [CD41] deposition, neutrophil infiltration, and inflammatory factors in the brain (39). A series of oxidative stress and inflammatory responses are the main pathological processes during cerebral ischemia/reperfusion (I/R) (124). PKM2 was upregulated after cerebral I/R injury in both *in vivo* middle cerebral artery occlusion (MCAO) or *in vitro* oxygen–glucose deprivation and reoxygenation (OGD/R) models of cerebral I/R injury. Knockdown of PKM2 improves neuronal cell viability and suppresses neuroinflammation, ultimately protecting neurological function from cerebral I/R damage (125). In addition, PKM2 knockdown significantly reduced the expression levels of oxidative stress factors (malondialdehyde (MDA), lactate dehydrogenase (LDH), and nitric oxide (NO)) (126). Knockdown of PKM2 expression levels has been reported to lead to growth inhibition and apoptosis of tumor cells (127). In non-tumor tissues, such as brain tissues subjected to hypoxia and ischemia, down-regulation of PKM2 expression also reduces the activity of the pro-apoptotic protein caspase-3, which inhibits the resulting apoptosis of neurons in the rat brain cortex (128). Mitochondrial dysfunction is a critical factor in the overdependence of neurons on glycolytic function during acute ischemia and/or reperfusion in ischemic stroke (129). Thus, protection of mitochondrial oxidative and autophagic functions, maintenance of mitochondrial homeostasis, and inhibition of glycolysis may play a key role in the brain tissue energy crisis during acute ischemia in AIS, versus the final extent of brain tissue injury after reperfusion (130).

### 5.4 PDK/PDH

The PDH complex catalyzes the conversion of pyruvate, the end product of glycolysis, to acetyl-coenzyme A (acetyl-CoA), which is further oxidized in the TCA cycle and ultimately utilizes mitochondrial OXPHOS to produce ATP (131). Therefore, the PDH complex serves as a gateway between glycolysis and the TCA cycle (132). The PDH complex consists of three major subunits: the PDH (E1), dihydrolipoic acid transacetylase (E2), and dihydrolipoic acid dehydrogenase (E3) (133). The activity of the PDH complex is tightly regulated by PDK, which phosphorylates serine residues on the *α*-subunit (E1α) of PDH, inhibiting its activity (134). This shift in pyruvate flux from OXPHOS to aerobic glycolysis is crucial in ischemic neurological disorders closely related to energy metabolism. Humans and rodents possess four PDK isozymes (PDK1-4), with PDK2 and PDK4 (PDK2/4) exhibiting a more generalized tissue distribution and being significantly associated with metabolic disorders compared to PDK1 and PDK3 (135, 136). Consequently, the PDK/PDH axis plays a pivotal role in ischemic neurological disorders.

Resting platelets primarily rely on OXPHOS and aerobic glycolysis to meet their energy demands (137). However, activated platelets require a high energy drive and exhibit a preferential glycolytic phenotype due to their limited mitochondrial density. This transition from a resting to an activated state is associated with an increased propensity for thrombosis (138). Arterial thrombosis, particularly in the carotid arteries, is a major contributor to the development and recurrence of ischemic stroke (139). The PDK/PDH axis has been shown to play a vital role in regulating aerobic glycolysis during the transition of platelets from a resting to an activated state. Combined genetic ablation of PDK2 and PDK4 inhibited platelet activation, reduced susceptibility to arterial thrombosis, and did not adversely affect hemostasis (60). Furthermore, deep vein thrombosis (DVT) is associated with an increased risk of ischemic stroke (140). The development of DVT requires continuous cooperation between neutrophils and platelets. The process of thrombosis triggers the formation of neutrophil extracellular traps (NETs), promoting platelet activation and intravascular coagulation (141). NET formation is an energy-intensive process that depends on aerobic glycolysis. Targeting PDK2/4 inhibits NET formation by reducing aerobic glycolysis, thereby further preventing DVT (61).

### 5.5 LHDA

LDH is an intracellular oxidoreductase-like enzyme that catalyzes the reversible conversion of pyruvate to lactate in the glycolytic pathway and the reversible conversion of NADH to NAD^+^ (63). LDH is encoded by four genes: *LDHA*, *LDHB*, *LDHC*, and *LDHD*. LDHA, LDHB, and LDHC are L isomers, while LDHD is a D isomer. The predominant enantiomer in vertebrates is the L isomer, which functions to use or produce L-lactic acid. Vertebrate L-LDHs are primarily composed of two identified structural isoforms: heart (H-type, encoded by LDHB) and muscle (M-type, encoded by LDHA) (142, 143). LDHB, which is more abundant in aerobic tissues, converts lactate to pyruvate, playing a significant role in lactate oxidation. When body tissues and cells consume large amounts of oxygen or experience inadequate oxygen supply (e.g., strenuous exercise, inflammation, ischemia, and tumors), the TCA cycle becomes inhibited, resulting in insufficient ATP production by OXPHOS. This activates the glycolytic pathway to compensate for the lack of ATP. During this process, lactate dehydrogenase (LDHA) reduces pyruvate and NADH to lactate and NAD^+^, respectively, to maintain the glycolytic pathway (144, 145).

HMGB1 was reported to be upregulated in both OGD/R-treated N2a cells and MCAO model rats. Meanwhile, Hgmb1 was found to be involved in the up-regulation of the histone H3 lysine 18 lactylation (H3K18lac) induced neuronal apoptosis by LDHA (146). The phenotype of microglia is regulated by LDHA. It was found that inhibition of LDHA promotes the release of anti-inflammatory factors (Arg1, Ym1, CD206) from microglia toward M2 polarization to exert anti-inflammatory effects, which in turn protects against rat cerebral I/R injury (147). Furthermore, a reduction in cerebral infarct volume 24 h after cerebral IR has been associated with the downregulation of LDHA expression in mice (148).

### 5.6 Lactate

Lactic acid, once mistakenly considered a “metabolic waste”, is now recognized as a crucial byproduct of glycolysis (149). Recent research has highlighted at least three significant roles for lactate. Firstly, lactate produced in active muscle tissue can be reused for glucose formation in the liver, completing the Cori cycle and enabling gluconeogenesis (149). Secondly, circulating lactate is a supplementary glucose source when blood glucose levels are insufficient to directly support neuronal activity. Moreover, while glucose primarily contributes indirectly to the TCA cycle, circulating lactate directly contributes to the production of TCA cycle intermediates, considered the primary energy source (150, 151). Thirdly, lactate can function as both an energy substrate and a signaling molecule, engaging in “lactate shuttling” (intra- or intercellular lactate exchange). The cytoplasmic (glycolysis)-mitochondrial (oxidative metabolism) exchange is a classic example of intracellular lactate shuttling. In the brain, the release of lactate from astrocytes to intercellular compartments via the monocarboxylic acid transporter protein (MCT)4 and its subsequent uptake by peripheral neurons through MCT2 for metabolism has been termed the astrocyte-neuron lactate shuttle (43, 152). Furthermore, a growing body of evidence supports lactate’s role as a signaling molecule that binds to and activates the specific receptor G protein-coupled receptor 81 (GPR81) for signaling (153–155).

Localized acute inflammatory responses or inadequate tissue perfusion are often accompanied by lactate accumulation (117). However, the contribution of lactate accumulation to ischemic brain injury remains controversial. Theoretically, lactate can function as an energy substrate during the ischemic phase, shuttling from astrocytes to neurons to provide energy and protect neuronal cells from ischemic damage. This hypothesis is supported by animal model studies (42, 156–159). However, the effect of lactate on nerve cells appears to be concentration-dependent. During cell culture, lower concentrations (5 mmol/L) of L-lactate had minimal effects on neuronal cell viability *in vitro*. At concentrations of 10 mmol/L, L-lactate produced results similar to the neuroprotection induced by MCT2-mediated lactate release through optogenetic stimulation of astrocytes (42). However, lactate concentrations below 10 mmol/L were insufficient to compensate for the energy crisis during cerebral ischemia. Even lactate concentrations up to 20 mmol/L only partially rescued the decreased ATP levels in N2A cells or primary neurons (160). Notably, higher concentrations (20 mmol/L) of L-lactate were neurotoxic, leading to an increase in apoptotic and dead cells (42, 157). The absence of partial pressure of oxygen (PO_2_) during the ischemic phase results in insufficient oxidative capacity to metabolize the accumulating lactate, which cannot be utilized as an energy substrate by neurons (161). Excessive lactate accumulation causes a significant decrease in cytoplasmic pH, leading to lactic acidosis (145, 162). This may be a more plausible explanation for the adverse effects of lactate accumulation. Additionally, recent evidence suggests that excessive lactate accumulation, particularly during ischemia, drives protein lactation (Kla) formation in mouse brain neurons, exacerbating brain injury (148). Therefore, while inhibiting lactate accumulation during ischemia might seem beneficial for neural recovery, excessive inhibition of glycolysis can lead to counterintuitive results. It has been found that during ischemia, when lactate accumulates at relatively low concentrations (approximately 1-3 mmol/L), lactate may exacerbate ischemic injury by activating GPR81 (160).

Studies have demonstrated that astrocyte activation induces the release of lactate into the extracellular space (42, 159). However, it cannot be ruled out that astrocytes may be activated through other mechanisms and subsequently participate in protecting neurons. Recently, some researchers have utilized *in vitro* cell cultures and animal models to observe that astrocyte lipoprotein receptor-related protein 1 (LRP1), a multifunctional transmembrane receptor, facilitates the transfer of healthy mitochondria from astrocytes to neurons by inhibiting glucose uptake, glycolysis, and lactic acid production, thereby preventing brain I /R damage. Furthermore, it was found that incubating astrocytes with graded doses of lactate (ranging from 0 to 50 mmol/L) for 2 h resulted in a dose-dependent decrease in extracellular mitochondria and ATP levels (163).

In conclusion, the spatiotemporal expression profile of lactate within the brain is dynamic and evolves in tandem with the progression of ischemic stroke. The impact of lactate on neuronal cells is multifaceted, encompassing both deleterious and protective effects, suggesting a complex regulatory mechanism. This regulation appears to be concentration-dependent, potentially accounting for the discrepancies observed in previous studies investigating the functional role of lactate.

## 6 Glycolysis-related molecular/signaling pathways in ischaemic stroke

**Figure 3:**
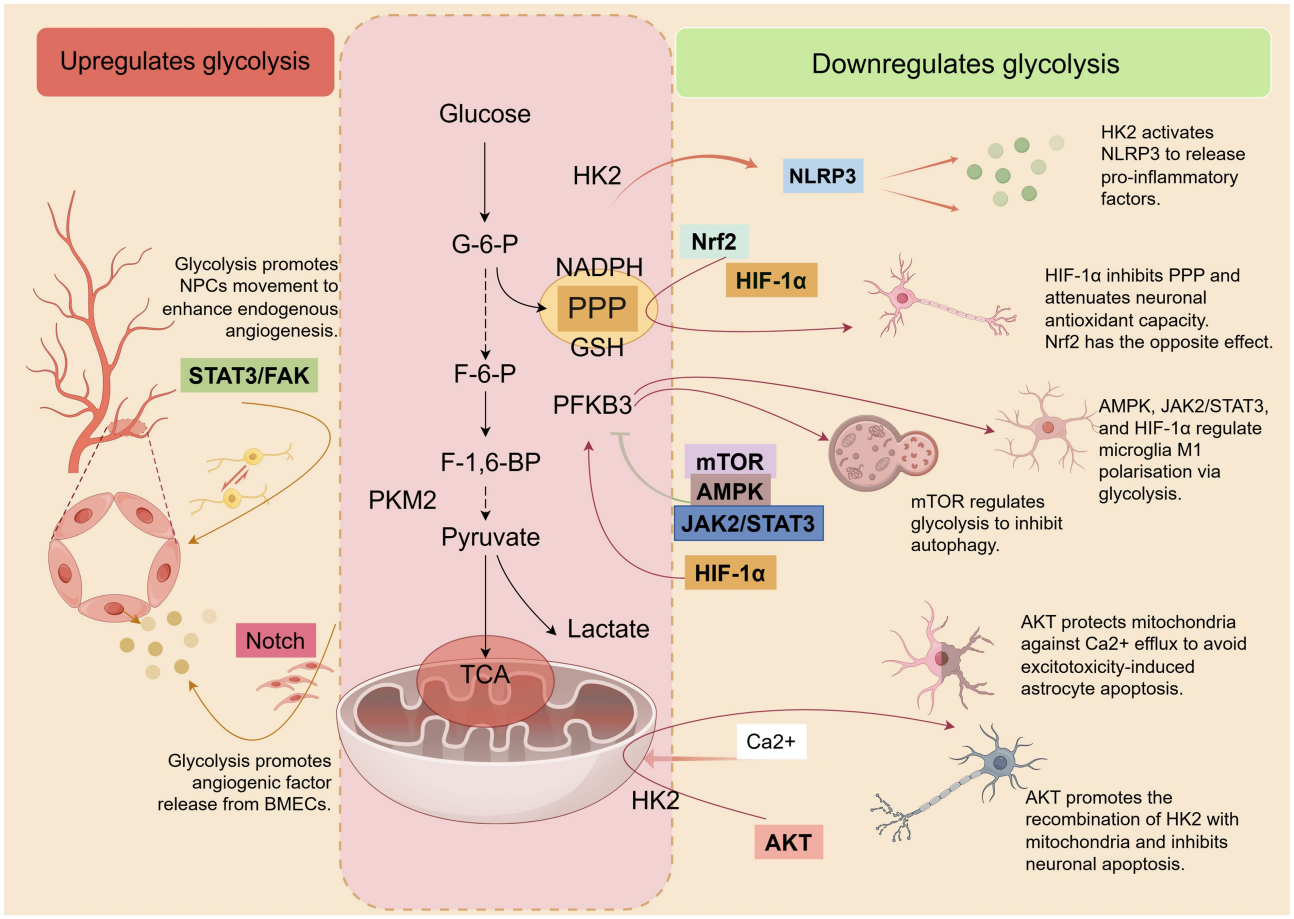
After the onset of IS, several molecules/signaling pathways involved in inflammation, oxidative stress, excitotoxicity, cell damage, and apoptosis are activated or inhibited. These pathways directly or indirectly regulate glycolysis and are also modulated by glycolysis, which in turn affects the pathological process of IS. Some of these pathways positively affect IS by decreasing glycolytic activity. Concurrently, there are also some pathways that have a positive effect on IS by elevating glycolytic activity. By Figdraw.

During the cerebral ischemia and/or reperfusion phase, the glycolytic pathway is overly relied upon as the primary mode of energy supply. This results in the activation or inhibition of a number of molecular/signaling pathways associated with the regulation of glycolysis, which are involved in neuroinflammation, oxidative stress, excitotoxicity, as well as cellular damage and apoptosis. Figure 3 summarises the contribution of glycolysis and related molecules/signaling pathways to IS.

The NACHT, LRR, and PYD domains-containing protein 3 (NLRP3) inflammatory vesicles are a complex of multiple proteins that play key roles in regulating the innate immune system and inflammatory signaling processes (164). Some studies have shown that when the NLRP3 inflammasome is activated, it releases downstream pro-inflammatory mediators such as IL-1β and IL-18, which become one of the key factors triggering neuroinflammation after cerebral ischemia–reperfusion (164, 165). In addition, glycolysis (mainly involving HK2 activity) is involved in the regulation of inflammatory vesicle activation and to play a crucial role in the activation of NLRP3 (166). An increase in glycolytic flux has been reported under hypoxic conditions, accompanied by a significant upregulation of HK1 and HK2. This leads to inhibition of the pentose phosphate pathway, reducing the conversion of pyruvate to acetyl coenzyme A, which in turn allows more pyruvate to be converted to lactate. In this environment, NLRP3 inflammatory vesicles are activated and release pro-inflammatory mediators, which trigger a neuroinflammatory response (167).

Adenosine monophosphate-activated protein kinase (AMPK) is a central regulator of cellular responses to energy metabolic stress. Its function is to sense changes in cellular energy status and maintain the balance between energy production and expenditure by regulating various metabolic and immune-related activities (168). AMPK has been reported to be an important protective factor against cerebral ischemic injury during cerebral ischemia or hypoxia (169). AMPK exerts an endogenous defense mechanism, possibly by promoting the reprogramming of energy metabolism in microglia (from glycolysis to OXPHOS) to enhance their immunophagocytosis and thus attenuate neuroinflammation and microglia M1 polarization in IS (51). A study found that the knockdown of protein kinase N1 (PKN1), a key brain developmental regulator, in an *in vivo* or *in vitro* model of stroke resulted in the improved coupling of the pyruvate and TCA cycles despite an increase in glycolytic fluxes, contributing to enhanced mitochondrial uptake of pyruvate. This helps to cope with energy metabolic stress and protects nerve cells from ischemic damage. However, when AMPK activation was inhibited by BAY-3827, the neuroprotective effect of PKN1 knockdown was counteracted. This suggests that in the absence of PKN1, activated AMPK exerts its protective effects more effectively in stroke models (170). Besides, studies have revealed the role of glycolysis in regulating cerebral angiogenesis after IS. This is mainly achieved through the activation of the key rate-limiting enzyme PFKFB3 as well as the signaling pathway represented by AMPK (171).

Mechanistic target of rapamycin (mTOR) is a key regulator of cellular metabolism, capable of integrating multiple signals from nutrients, growth factors, energy status, and oxygen levels. mTOR forms two protein complexes, mTORC1 and mTORC2, but mTORC1 signaling has broader effects on the growth and homeostasis of key groups of immune cells and organs (172, 173). It was found that downregulation of PFKFB3-driven glycolysis partially reduced lactate-mediated activation of mTORC1, thereby inhibiting MDSC differentiation to mature inflammatory cells to attenuate acute ischemic brain injury (95). Autophagy is activated after I/R, but there is still debate on whether autophagy activation is a pro-survival or pro-death mechanism in IS (174). It has been shown that TP53-induced glycolysis and apoptosis regulator (TIGAR) reduces I/R-induced autophagic activity in mouse brain and primary cortical neurons in both *in vivo* and *in vitro* experiments. In addition to the possible mechanism of ROS reduction, TIGAR inhibits autophagy by regulating the mTOR-S6KP70 signaling pathway, thereby preventing neuronal damage (174).

Signal transducer and activator of transcription 3 (STAT3) is important for vertebrate development and the functional performance of mature tissues. It is also involved in the regulation of inflammatory and immune responses (175). Overexpression and/or aberrant activation of the STAT3 signaling pathway has been previously observed in a variety of human diseases, such as cancer, autoimmune diseases, and inflammatory diseases (176). Recent studies have found that glycolysis can increase inflammatory responses to cerebral ischemia by up-regulating the phosphorylation of STAT3, which in turn enhances the infiltration of peripheral blood neutrophils and the expression of inflammatory cytokines (39). Janus kinase 2 (JAK2) is expressed in a variety of tissues and cell types, and it has important effects on cell differentiation, apoptosis, and immune regulation. The JAK2/STAT3 signaling pathway is a classical signaling module that plays a key role in several aspects of the mammalian immune system (177). It was found that both JAK2/STAT3 expression was dramatically increased in microglia treated with OGD/R *in vitro*. By blocking the JAK2/STAT3 signaling pathway, the OGD/R-induced aerobic glycolysis process in microglia could be reduced and its polarization toward M2-type could be promoted (178). When the stroke occurs, astrocytes normally produce protective mechanisms for neurons (ANLS, mitochondrial transmission, and antioxidant support as mentioned above in this review). However, the mechanism of astrocyte-mediated neurotoxicity after stroke has also been reported recently. In an OGD/R-treated astrocyte-neuron indirect co-culture system, astrocytes in an ischemic state activate the STAT3 signaling pathway, which leads to an upregulation of the lactate-directed glycolytic process and is accompanied by impairment of mitochondrial function. These damaged mitochondria are delivered to healthy neurons by astrocytes, which may be a potential mechanism of toxicity leading to synaptic degeneration in nonischemic neurons. However, if STAT3 inhibition in ischemic astrocytes prevents neuronal synaptic degeneration, this may be due to the restoration of mitochondrial function in astrocytes (45). Focal adhesion kinase (FAK) is a regulator involved in cell survival, proliferation, and migration (179). Activation of the STAT3/FAK signaling pathway promotes NPCs migration and enhances endogenous angiogenesis and neurogenesis in ischemic brain injury (180). This suggests that STAT3 activation after cerebral ischemia is not the only negative effect of neural tissue damage.

Hypoxia inducible factor-1α (HIF-1α) is a is a sensitive regulator of oxygen homeostasis. It regulates endogenous adaptive programs such as glycolytic pathways, inflammatory responses, and oxidative stress by modulating multiple signaling pathways and targeting downstream genes under hypoxic conditions (181, 182). HIF-1α expression is rapidly induced when tissues or cells are hypoxic/ischemic (182). It was found that when microglia converge to the M1 phenotype during AIS, the shift of OXPHOS to aerobic glycolysis becomes a prominent energy metabolism feature, a process that is dependent on the activation of the AMPK/mTOR/HIF-1α signaling pathway (183). In addition, numerous studies revealed that HIF-1α significantly increased the glycolytic flux of microglia by mediating the activity of glycolysis-related enzymes such as HK2, PKM2, and LDHA, which promoted their conversion to the M1 phenotype and triggered neuroinflammation (59, 147, 184–186). It is worth mentioning that activation of HIF-1α during AIS may help to attenuate nerve tissue damage and dysfunction induced by ischemia, but this depends on the different types of nerve cells. A recent study found that timely activation of HIF-1α in astrocytes in the early stages of ischemia and enhancement of ischemic tolerance in astrocytes can improve the prognosis of acute ischemic stroke. However, if HIF-1α is overactivated in neurons, it triggers a HIF-1α-dependent increase in PFKFB3 expression, leading to a shift of F-6-P from the PPP pathway to the glycolytic pathway. This process is accompanied by a decrease in NADPH production in the PPP pathway, which in turn weakens the already low antioxidant defenses of neurons and makes them more vulnerable to damage from ischemic stress. Interestingly, in an environment where neurons are co-cultured with astrocytes, the sensitivity of neurons to ischemic stress triggered by hyperactivation of HIF-1α is reduced. This may be due to the strong antioxidant support that neurons receive from their surrounding astrocytes (55).

Nuclear factor-E2-related factor 2 (Nrf2) is an important transcription factor for the maintenance of redox homeostasis, which regulates cellular defenses against toxicity and oxidative damage through the expression of genes involved in oxidative stress response and drug detoxification (187). The study revealed that aerobic glycolysis was significantly enhanced during I/R, while the activity of antioxidant enzymes was inhibited. This process leads to impaired mitochondrial function and the release of large amounts of reactive oxygen species (ROS), which in turn disrupts the blood–brain barrier and exacerbates brain damage. However, by activating downstream antioxidant proteins associated with Nrf2, oxidative metabolic homeostasis can be restored, blood–brain barrier integrity can be protected, and neuronal death can be reduced (14). NADPH provided by the PPP pathway plays a crucial role in the defense against ischemia-induced oxidative stress in neurons. TP53-induced glycolysis and apoptosis regulator (TIGAR) was found to activate Nrf2 in ischemic neurons by promoting autophagic processes, thereby shifting glucose metabolism to the PPP pathway to reduce oxidative stress and prevent ischemic neuronal injury (188).

The kinase AKT (protein kinase B), is a key signaling molecule that regulates cell survival, proliferation, tumor development, and angiogenesis (189). The diminished activity of AKT is strongly associated with cellular metabolic disorders and apoptosis (190). It was found that a mechanism dependent on AKT (p-AKT) inactivation induced neuronal apoptosis after PKM2 upregulation during brain tissue ischemia and hypoxia (128). Cerebral ischemia triggers neuronal cell apoptosis through multiple mechanisms, of which mitochondrial dysfunction is particularly critical. Glutamate excitotoxicity leads to the dissociation of HK2 from mitochondria, which in turn disrupts the structural and functional integrity of mitochondria. This process is manifested by the opening of the mitochondrial permeability transition pore, the collapse of the mitochondrial membrane potential, and a decrease in the neuronal mitochondrial oxygen consumption ratio, which ultimately leads to apoptosis of the neuronal cell. However, promoting the recombination of HK2 with mitochondria through the activation of AKT and protecting HK activity by improving the glycolytic process can effectively protect neurons from apoptosis (191). Although astrocytes exhibit a greater ability to tolerate ischemic and hypoxic environments than neurons, exposure of astrocytes to acidic pH (6.8, 6.0) environments during ischemia when the glycolytic product lactate accumulates to a certain level leads to the release and inward flow of Ca2+, which in turn triggers mitochondrial hyperpolarization, ultimately leading to astrocyte death. This process is accompanied by activation of p38 MAPK and inhibition of AKT. However, SC79, an AKT activator, prevents mitochondrial hyperpolarization in astrocytes (192). Activation of AKT is usually achieved through close association with activated kinases of PI3K and is involved in the regulation of glycolysis-related enzymes (193). However, there seems to be a lack of studies on the effects of neurocellular glycolysis and cell survival after stroke via the PI3K/AKT pathway, suggesting that further exploration may be needed in the future.

In addition to the above, several pathways related to the regulation of glycolysis in IS have been reported. For example, the Wnt/*β*-catenin pathway, which has recently been reported to be involved in IS progression by affecting the several enzymes of glycolysis, NADPH/NADP, and the levels of ATP, induces ferroptosis of tract astrocytes (194). Notch signaling pathway (closely related to angiogenesis) can be activated by PFKFB3 to promote the formation of brain microvascular endothelial cells (BMECs) after stroke (195). AK4 enhances neuronal adaptive responses to hypoxia/ischemia by promoting Parkin-mediated PKM2 degradation (130). Knockdown of PKM2 increases neuronal cell viability and inhibits inflammatory cytokines (IL-1β, IL-6, and TNF-*α*) released by HMGB1-mediated TLR4/MyD88/TRAF6 expression, ultimately protecting neurological function from cerebral I/R damage (125).

## 7 Diagnostic potential of glycolysis in ischemic stroke

Glycolytic metabolic processes, particularly enzymes like HK2, PFKFB3, PKM2, PDH, LHD, and metabolites such as lactate, have emerged as potential predictors of stroke risk, development, and prognosis (39, 59, 196–199). This suggests their potential diagnostic value. HK2, specifically expressed in microglia, is upregulated in activated microglia and tightly controls glycolytic flux. Assessing HK2 levels can dynamically evaluate microglial glycolysis and bioenergetic responses, making it a promising marker for early diagnosis of IS (72). Neutrophils, among the first responders to ischemic injury (121, 122). Exhibit elevated levels of nuclear PKM2 in ischemic stroke patients treated with mechanical thrombolysis compared to healthy controls (39). This suggests that continuous monitoring of PKM2 marker levels could reflect clinical outcomes after thrombolysis, though further studies are needed. Additionally, research has implicated the fatty acid-related PPAR signaling pathway and PKM2 as key markers of neuronal cells responding to OGD/R, potentially aiding in early diagnosis (200). The pathogenesis of IS involves significant immune infiltration and the participation of multiple genes. PFKFB3, identified as a core functional gene through clinical sample validation, has emerged as a potential early diagnostic marker for ischemic stroke (201).

Conventional clinical tools such as computed tomography (CT) and magnetic resonance imaging (MRI) are currently employed to diagnose IS. While these methods enable rapid assessment of infarct location and volume, they are less effective in determining the underlying cause of infarcts (202). A dearth of research on imaging biomarkers that reflect dynamic changes in ischemic brain tissue has hindered the ability to sensitively assess tissue viability within the ischemic penumbra (199). Recent investigations have demonstrated the potential of hyperpolarized [1-^13^C]pyruvate as an imaging biomarker to characterize changes in tissue viability during reperfusion and subsequent brain tissue recovery (203). By monitoring the conversion of hyperpolarized [1-^13^C]pyruvate to [1-^13^C]lactate and [^13^C]bicarbonate, researchers have quantified the rates of LDH and PDH enzymes. The results revealed a significant increase in the LDH/PDH enzyme activity ratio during IR, followed by a decrease to pre-ischemic levels during recovery. These findings suggest that this ratio could serve as a potential indicator for detecting changes in IS disease (199).

Glycolysis-related enzymes and metabolites, including LDH, lactate, and pyruvate, have been observed at elevated levels in the plasma and cerebrospinal fluid (CSF) of IS patients, indicating active glycolysis (204–206). AIS is often accompanied by impaired consciousness, the underlying causes can also be attributed to non-structural brain tissue changes, such as intoxication, infection, or metabolic disorders (204). CSF-LDH values have been found to differentiate between structural and non-structural etiologies in cases of acute alterations in consciousness, especially in the absence of trauma or access to conventional imaging tools (204). This holds promise for providing a reliable diagnostic measure in regions with limited medical resources. In a study involving 5,129 patients with AIS, high LDH levels were associated with an increased risk of dysfunction and recurrence, partially explained by stroke recurrence (207). Another multicenter retrospective study of 527 AIS patients revealed that elevated serum LDH levels during the first 3 days after intravenous thrombolytic therapy were strongly predictive of adverse clinical outcomes, including hemorrhagic conversion, neurological deterioration, disability, and others, 3 months post-thrombolysis (196). Additionally, LDH levels have been shown to predict the risk of DVT during rehabilitation in stroke patients (208). LDH demonstrates significant potential in predicting the risk of IS recurrence and clinical outcomes. Among thrombolytic adverse outcomes, hemorrhagic conversion poses the most significant limitation to the clinical use of rt-PA. The endogenous metabolite lactate has been identified as a potential biomarker for early prediction of hemorrhagic conversion (198). Overall, these biomarkers offer the potential to improve diagnostic accuracy, predict the risk of adverse outcomes, and inform clinical decision-making (Table 1).

**Table 1:** Diagnosis of IS by glycolysis.

IS pathology/clinical stage	Sample	Marker	Significance	Reference
Ischemic phase of brain tissue	Mouse microglial cells	HK2	Assist in the early diagnosis of IS	(72)
After IR	Human brain-like organ	PKM2	Assist in the early diagnosis of IS	(200)
After mechanical thrombolysis	Human peripheral neutrophils	PKM2	Correlation with stroke outcome after reperfusion	(39)
Stages of inflammatory reaction in brain tissue	Human serum	PFKFB3	Assist in the early diagnosis of IS	(201)
After IR	Brain sections of rats	LDH/PDH	Tissue response to different pathological stages	(199)
Before the onset of the disease	Human plasma or serum	Pyruvate	Associated with increased risk of IS	(206)
During acute alterations in the level of consciousness	Human cerebrospinal fluid	LDH	Distinguishes between structural and nonstructural etiologies	(204)
During post-stroke recovery	Human serum	LDH	Predicts risk of recurrence and adverse clinical outcome	(207, 208)
After intravenous thrombolysis with rt-PA	Human serum	LDH	Predicts adverse clinical outcomes after thrombolysis	(196)
After intravenous thrombolysis with rt-PA	Rat cerebral cortex and serum	Lactate	Predicts risk of hemorrhagic transformation after thrombolysis	(198)

## 8 Pharmacotherapeutic applications of glycolysis in ischemic stroke

Beyond accurate diagnosis, precise treatment is paramount in improving the prognosis of IS and preventing potentially severe complications. Glycolytic metabolic pathways are gaining recognition as promising therapeutic targets for neurological disorders, not only in tumor cells but also across multiple cell types.

AZ67, a potent and selective inhibitor of PFKFB3, has been shown to protect neurons from excitotoxicity following IR by inhibiting PFKFB3 activity (54). Pretreatment of mice with oxalate, an LDHA inhibitor, has been reported to significantly reduce neuronal death and infarct volume 24 h after reperfusion in ischemic stroke rats (148). Natural bioactive products, known for their high efficacy and safety compared to enzyme inhibitors, have been increasingly studied for inflammation-related diseases. Tetrahydroxy stilbene glucoside (TSG), a small molecule natural bioactive ingredient, has been shown to reverse the energy metabolism profile of M1 macrophages to the M2 state, exerting potent anti-inflammatory and neuroprotective properties. This effect of TSG is closely linked to the conformational change of PKM2 (209). Benzoxepane derivatives 10a exhibit anti-inflammatory effects both *in vitro* and *in vivo* by inhibiting PKM2-mediated glycolysis and NLRP3 (an inflammation-activating protein) activation, leading to beneficial effects on ischemic stroke (210).

Chlorpromazine and promethazine (C + P) can induce depressive or hibernation-like effects on brain activity, leading to pharmacological hypothermia, which reduces HIF-1α and glycolytic enzyme (PFK-1, LDH) levels and promotes neuroprotection after ischemic stroke (211). Combining dihydrocapsaicin (DHC) with C + P not only inhibits glycolytic and gluconeogenic enzyme activities but also reduces neurological damage caused by oxidative stressors, such as ROS and NADPH oxidase (NOX) (212). However, the most problematic side effect of pharmacological hypothermia is the risk of increased blood viscosity at low temperatures, leading to platelet aggregation and potential microcirculatory obstruction in the brain and heart (213). Intravenous administration of ethanol (1.0 g/kg) has been reported to achieve similar hyperglycolytic inhibition with pharmacological hypothermia (33°C), suggesting a potentially more straightforward and safer treatment option after stroke (213). Arginine, an essential amino acid with anti-inflammatory properties, has been shown to reduce HIF-1α/LDHA-mediated neuronal death and improve functional recovery in stroke animals after brain IR injury when injected into the ventricles (147). Leptin, administered via tail vein injection in mice after transient ischemia, has been shown to significantly reduce LDH levels, the lactate/pyruvate ratio, neurological deficits, infarct volume, and cerebral edema (214). Dichloroacetate (DCA), a small molecule that activates PDH and crosses the blood–brain barrier (BBB), reduces neurotoxic lactic acidosis during cerebral ischemia and protects BBB integrity (14, 215, 216). Recently, researchers have synthesized fasudil dichloroacetate (FDCA), a novel drug that combines the mechanisms of action of fasudil and dichloroacetate, resulting in superior efficacy in reducing cerebral ischemic injury compared to their individual or combined use (217). Celastrol is a naturally occurring bioactive compound that inhibits glycolysis via the HIF-1α/PDK1 axis and provides neuroprotection in cerebral I/R injury (218).

Targeted regulation of key glycolytic enzymes by active ingredients from traditional Chinese medicine represents a promising approach for the effective prevention and treatment of IS in the future. Regulatory T cells (Tregs), crucial regulators of inflammation, play a vital role in maintaining immune tolerance and homeostasis (219). *Ginkgo biloba* extract (EGb) has been shown to protect neurons from inflammatory damage by inhibiting the HIF-1*α*/HK2 signaling pathway and promoting the proliferation and differentiation of Tregs (184). Salvianolic acid C (SAC), an active ingredient in *Salvia miltiorrhiza*, inhibits the M1 polarization of microglia through the glycolytic pathway (down-regulation of PKM2), reducing the expression of inflammatory factors TNF-α, IL-6, and IL-1β, and protecting against neurological injury in a MCAO model (220). Panax notoginseng saponins (PNS) administration has been found to inhibit microglia activation-induced HIF-1α/PKM2/STAT3 signaling activation, counteracting ischemic brain injury (221). Icariin has been reported to inhibit neuronal cell inflammation and apoptosis by down-regulating PKM2 overexpression after OGD in neuronal cells (222). Baicalin improves PDK2-PDH function during IR by inhibiting succinate dehydrogenase (SDH)-mediated oxidative stress and increasing pyruvate flow to the mitochondria, supplying neurons with sufficient energy rather than relying on aerobic glycolysis (132). In addition, ginsenoside Rd. (Rd) likewise increases pyruvate flow to mitochondria, reducing the release of cytochrome c (CytoC) and apoptosis-inducing factor (AIF) from mitochondria, thus maximizing the inhibition of neuronal apoptosis (223). Astragaloside IV (AIV), an active ingredient in Astragalus, helps to counteract ischemia-induced neuronal apoptosis and DNA damage by activating the Akt signaling pathway, protecting mitochondrial HK2, and reducing the release of pro-apoptotic proteins and AIF (191). AIV was able to regulate the expression of key glycolytic enzymes (HK2, PFKFB3, PKM2) and energy transporter proteins (GLUT1, MCT1, MCT4) through activation of the AMPK pathway and inhibition of the mTOR/HIF-1α pathway, which promotes microglial cell polarization toward the M2-type and inhibits M1-type polarization (224). Upregulation of glycolysis after IS onset does not necessarily imply a negative effect on brain tissue recovery. BMECs have been reported to rely primarily on glycolytic function, whereas other vascular cells rely on aerobic respiration (225). Hydroxysafflor Yellow A (HSYA), a major bioactive constituent of saffron, was found to enhance angiogenesis in BMECs after OGD/R by promoting glycolysis (195). Moreover, classical Chinese herbal remedies, which have the advantage of multi-target and multi-pathway regulation, have also been applied in the treatment of IS. Taohong Siwu Decoction (THSWD) can accelerate mature angiogenesis by promoting the expression of mature angiogenic factors (VEGFA, Ang1, and PDGFB) through the activation of glycolysis (225). Buyang Huanwu decoction (BHD) is a classic prescription for promoting angiogenesis after IS by maintaining moderate levels of glycolysis in ischemic brain tissue, as well as promoting angiogenesis (171). Mailuo Shutong Pill (MLST) suppresses brain tissue inflammation by modulating glucose metabolism disorders, interfering with immunometabolic reprogramming, inhibiting microglia infiltration, and down-regulating the NLRP3 inflammatory vesicle signaling pathway, ultimately ameliorating cerebral ischemia–reperfusion injury (167) (Table 2).

**Table 2:** Treatment of IS by glycolysis.

Drug	Model	Target/pathway	Effect	Reference
EGb	MCAO&Mouse CD4 + CD62L+ T cells	HIF-1α/HK2	Inhibition of glycolysis promotes Treg cell differentiation	(184)
AIV	MCAO&OGD-treated primary cortical neurons	Akt/HK2	Protection of mitochondrial integrity Inhibition of apoptosis	(191)
	MCAO&*In vitro* mouse BV2 cells	AMPK/mTOR/HIF-1α/glycolysis	Inhibition of microglia M1 differentiation	(224)
AZ67	MCAO&Cortical neurons of mice treated with OGD/R	PFKFB3	Prevention of apoptotic cell death induced by neuronal excitotoxic stimuli and OGD/R	(54)
BHD	MCAO	AMPK/PFKFB3	Maintaining moderate levels of glycolysis in brain tissue Promotion of angiogenesis	(171)
TSG	BCCAO&*In vitro* mouse bone marrow-derived macrophages (BMDMs)	PKM2	Regulation of PKM2 conformation to influence macrophage plasticity	(209)
10a	LPS-induced neuroinflammatory mouse&MCAO&*In vitro* mouse BV2 cells	PKM 2/NLRP3	Inhibition of glycolysis and NLRP3 activation	(210)
SAC	MCAO&*In vitro* mouse BV2 cells	PKM2	Inhibition of M1 polarization of BV2	(220)
PNS	Photothrombotic stroke model	HIF-1α/PKM2/STAT3	Inhibition of microglia activation and inflammation	(221)
Icariin	OGD-treated mouse neuroblastoma N2a cells	PKM2	Reduced levels of pro-apoptotic proteins (caspase 3 and Bax)	(222)
Celastrol	MCAO	HIF-1α/PDK1	Inhibition of glycolysis Prevention of cerebral I/R damage	(218)
Baicalin	OGD/R-treated mouse neuroblastoma N2a cells&MCAO/R	PDK2-PDH	Inhibition of SDH-mediated oxidative stress improves neuronal glucose metabolism	(132)
DCA	MCAO/R&Human brain microvascular endothelial cells (HBMEC) *in vitro*	PDK2-PDH-Nrf2	Reduction of lactate accumulation after IR injury, inhibition of oxidative stress and protection of blood–brain barrier integrity	(14, 216)
FDCA	MCAO&OGD-treated mouse brain endothelial cells (bEnd3)	PDK1-PDH	Inhibition of PDH phosphorylation to reduce lactate release	(217)
Oxalate	MCAO	LDHA	Inhibition of glycolytic pathway or lactic acid production	(148)
Arginine	MCAO&Microglia and neurons treated with OGD/R	HIF-1α/LDHA	Inhibits the pro-inflammatory effects of microglia	(147)
Leptin	MCAO&OGD/R-treated human neuroblastoma (SH-SY5Y)	p-Akt/LDH	Decreases lactate/pyruvate ratio and increases brain glucose uptake and ATP levels.	(214)
C + P	MCAO&OGD/R-treated human neuroblastoma (SH-SY5Y)	Glycolysis	Regulates HIF-1α and attenuates excessive glycolysis and NOX activation	(211)
C + P + DHC	MCAO/R	Glycolysis	Inhibition of stroke-induced dysfunctional hyper glycolysis and gluconeogenesis	(212)
Ethanol	MCAO/R	Glycolysis	Inhibition of increased glucose utilization and lactate production after stroke	(213)
Rd	MCAO&*in vitro* Nonsynaptosomal mitochondria	Glycolysis	Increase of pyruvate flow to mitochondria Inhibition of apoptosis in neuronal cells	(223)
HSYA	OGD/R-treated mouse BMECs	Glycolysis	Promotion of migration and angiogenesis of BMECs by glycolysis	(195)
THSWD	MCAO/R&OGD/R-treated mouse BMECs	Glycolysis	Activation of the glycolytic pathway promotes mature angiogenesis	(225)
MLST	MCAO/R	Glycolysis/NLRP3	Inhibition of the level of inflammation in brain tissue	(167)

## 9 Conclusions and future perspectives

Overall, modulation of glycolytic processes in immune cells, neuronal cells, and endothelial cells through multiple pathways to influence the onset and progression of IS has become a relatively well-established research strategy. However, we seem to have some previous uncertainty as to whether glycolysis should be promoted or inhibited in IS. However, after careful combing of the literature, we found that among the key cells involved in the pathomechanisms of IS, inhibition of glycolytic activity resulted in many cells exhibiting positive effects on brain tissue recovery. These cells include monocytes/macrophages, neutrophils, platelets that promote inflammation in arterial plaques and thrombi, and microglia that induce neuroinflammation. However, in vascular endothelial cells, increased glycolytic flux contributes to the promotion of neovascularization, which also has a positive effect on brain tissue recovery in IS. Interestingly, in tumor tissues, elevated glycolytic activity of vascular endothelial cells tends to lead to overgrowth of tumor tissues and, therefore, its activity needs to be inhibited (46). It follows that the variability of the functions played by glycolysis in different disease environments as well as in cells of different tissues determines whether its up-regulation is beneficial or not. However, it is important to note that the current study has not clarified the extent to which glycolysis is up- or down-regulated in different cells, which may require further exploration in the future. In related studies, the development and prognosis of the disease can be predicted by monitoring the levels of glycolysis-related enzymes as well as metabolites.

Monitoring glycolysis-related enzymes and metabolites can aid in predicting disease development and prognosis. Targeting these enzymes and metabolites has shown potential in reducing neuronal damage and promoting neuronal function recovery in both experimental and clinical settings. However, the study of glycolysis and IS faces several limitations: (1) The mechanisms underlying glycolysis and IS are complex, involving intricate regulatory interactions among neurons, microglia, and astrocytes. A comprehensive understanding requires a broader perspective beyond the effects of glycolysis on microglial immunosurveillance or astrocyte lactate delivery. (2) Assessing reactive glycolytic activity can be challenging due to the limitations of existing indices. Lactate accumulation may not accurately reflect glycolytic activity, as other pathways (e.g., glutamine and pentose phosphate pathways) also contribute to lactate production. (3) The lack of uniformity in animal models, cellular models, and interventions can lead to inconsistent or even contradictory results for the same enzyme, as the expression of the same enzyme in different cell types is often specific. (4) The use of glycolysis-related enzymes and metabolites as diagnostic indicators remains relatively broad, lacking objective, precise, and specific quantitative criteria. (5) The primary use of intraperitoneal injection in animal models may not fully represent the clinical administration route, requiring further investigation to determine the relevance of the findings to the actual clinical setting. Future research can address these limitations by (1) Considering the complex intercellular functions and regulatory mechanisms to avoid one-sided conclusions. (2) Standardizing and unifying experimental modeling, intervention methods, and observation indices to ensure cell specificity and minimize interference from other factors. (3) Providing detailed descriptions of the trends in index changes and corresponding disease stages for diagnostic purposes. (4) Exploring intranasal administration as a promising drug delivery method for treating IS, offering advantages such as convenience, non-invasiveness, bypassing the blood–brain barrier, rapid delivery to the brain and spinal cord, minimal systemic exposure, and reduced side effects (226, 227). In conclusion, both diagnosing/predicting IS through glycolysis and treating IS by targeting glycolytic pathways represent promising research approaches that warrant further in-depth investigation in the context of ischemic stroke.
